# Short- and Medium-Term Efficacy of a Web-Based Computer-Tailored Nutrition Education Intervention for Adults Including Cognitive and Environmental Feedback: Randomized Controlled Trial

**DOI:** 10.2196/jmir.3837

**Published:** 2015-01-19

**Authors:** Linda Springvloet, Lilian Lechner, Hein de Vries, Math JJM Candel, Anke Oenema

**Affiliations:** ^1^Maastricht UniversityDepartment of Health Promotion, School for Public Health and Primary Care (CAPHRI)MaastrichtNetherlands; ^2^Open University of the NetherlandsFaculty of Psychology and Educational SciencesHeerlenNetherlands; ^3^Maastricht UniversityDepartment of Methodology and Statistics, School for Public Health and Primary Care (CAPHRI)MaastrichtNetherlands

**Keywords:** cognitive feedback, environmental feedback, self-regulation, computer tailoring, nutrition education, fruit consumption, vegetable consumption, fat consumption, snack consumption

## Abstract

**Background:**

Web-based, computer-tailored nutrition education interventions can be effective in modifying self-reported dietary behaviors. Traditional computer-tailored programs primarily targeted individual cognitions (knowledge, awareness, attitude, self-efficacy). Tailoring on additional variables such as self-regulation processes and environmental-level factors (the home food environment arrangement and perception of availability and prices of healthy food products in supermarkets) may improve efficacy and effect sizes (ES) of Web-based computer-tailored nutrition education interventions.

**Objective:**

This study evaluated the short- and medium-term efficacy and educational differences in efficacy of a cognitive and environmental feedback version of a Web-based computer-tailored nutrition education intervention on self-reported fruit, vegetable, high-energy snack, and saturated fat intake compared to generic nutrition information in the total sample and among participants who did not comply with dietary guidelines (the risk groups).

**Methods:**

A randomized controlled trial was conducted with a basic (tailored intervention targeting individual cognition and self-regulation processes; n=456), plus (basic intervention additionally targeting environmental-level factors; n=459), and control (generic nutrition information; n=434) group. Participants were recruited from the general population and randomly assigned to a study group. Self-reported fruit, vegetable, high-energy snack, and saturated fat intake were assessed at baseline and at 1- (T1) and 4-months (T2) postintervention using online questionnaires. Linear mixed model analyses examined group differences in change over time. Educational differences were examined with group×time×education interaction terms.

**Results:**

In the total sample, the basic (T1: ES=–0.30; T2: ES=–0.18) and plus intervention groups (T1: ES=–0.29; T2: ES=–0.27) had larger decreases in high-energy snack intake than the control group. The basic version resulted in a larger decrease in saturated fat intake than the control intervention (T1: ES=–0.19; T2: ES=–0.17). In the risk groups, the basic version caused larger decreases in fat (T1: ES=–0.28; T2: ES=–0.28) and high-energy snack intake (T1: ES=–0.34; T2: ES=–0.20) than the control intervention. The plus version resulted in a larger increase in fruit (T1: ES=0.25; T2: ES=0.37) and a larger decrease in high-energy snack intake (T1: ES=–0.38; T2: ES=–0.32) than the control intervention. For high-energy snack intake, educational differences were found. Stratified analyses showed that the plus version was most effective for high-educated participants.

**Conclusions:**

Both intervention versions were more effective in improving some of the self-reported dietary behaviors than generic nutrition information, especially in the risk groups, among both higher- and lower-educated participants. For fruit intake, only the plus version was more effective than providing generic nutrition information. Although feasible, incorporating environmental-level information is time-consuming. Therefore, the basic version may be more feasible for further implementation, although inclusion of feedback on the arrangement of the home food environment and on availability and prices may be considered for fruit and, for high-educated people, for high-energy snack intake.

**Trial Registration:**

Netherlands Trial Registry NTR3396; http://www.trialregister.nl/trialreg/admin/rctview.asp?TC=3396 (Archived by WebCite at http://www.webcitation.org/6VNZbdL6w).

## Introduction

Promoting healthy diets remains an important public health target because unhealthy dietary intake patterns are highly prevalent in most Western countries [1-3]. Of Dutch adults, only 3%-14% comply with the guideline of consuming 200 grams of vegetables a day and only 4%-26% comply with the guideline of 2 pieces of fruit a day [4]. Furthermore, 88%-92% have a higher habitual intake of saturated fat than the recommended 10 energy percent (E%) [4]. Because high-energy snacks often contain a lot of saturated fat [5], decreasing high-energy snack intake could result in a considerable decrease in the intake of saturated fat. The intake of fruit, vegetables, and saturated fat is even more unfavorable among lower-educated people compared to higher-educated people [4,6-8], making this an important target group for nutrition education interventions. Not complying with dietary guidelines is an important risk factor for multiple chronic diseases, such as cancer, cardiovascular diseases (CVD), and type 2 diabetes mellitus [9]. Therefore, it is important to improve the intake of fruit, vegetables, high-energy snacks, and saturated fat, especially among people who do not comply with dietary guidelines (ie, risk groups).

To modify dietary intake patterns in large population groups, intervention techniques that can reach large numbers of people and that can be tailored to individual dietary intake patterns are required. Computer tailoring is a suitable technique that can reach a large number of people at relatively low cost [10]. In computer-tailored nutrition education, health information is adapted to the specific needs and characteristics of a person [11,12]. Several reviews have shown that Web-based computer-tailored interventions can be effective in improving the self-reported intake of fruit, vegetables, and fat compared to generic or no information [10,13-15], also among lower-educated people [16,17]. The effect sizes (ES) of existing computer-tailored nutrition education interventions are, however, often small [10,13,15]. Therefore, it is important to find ways to increase the size of the effects, (eg, by targeting “new” determinants or behavior change processes). Until now, nutrition education interventions have primarily targeted motivational determinants, such as attitude and self-efficacy. Although motivation is an important first step in the behavior change process, it is not likely that motivation alone will lead to sustained behavior change [18-20]. This approach neglects important volitional [18,20] and self-regulation processes, such as goal setting and action planning, which focus on translating intention into action and facilitate actual changes.

Self-regulation skills, such as planning and monitoring, are shown to predict dietary behavior [21]. Using intervention techniques that foster self-regulation, such as goal setting and providing feedback on performance, is associated with larger improvements in dietary outcomes [22]. In addition, a meta-regression by Michie and colleagues [23] showed that including the self-regulatory technique self-monitoring of behavior in combination with other self-regulation behavior change techniques (ie, prompt intention formation, prompt specific goal setting, provide feedback on performance, and prompt review of behavioral goals) is likely to increase the efficacy of interventions aimed at promoting healthy nutrition. Self-regulation processes such as goal setting and action planning were shown to be feasible to apply in interventions targeting weight maintenance [24], weight loss among young adults [25], and diabetes management [26], but the additional effect of targeting self-regulation processes in computer-tailored interventions has yet to be established.

In addition to individual-level factors, environmental-level factors may play a role as drivers of behaviors [27,28]. The evidence regarding environmental-level determinants for dietary behaviors is, however, not compelling yet and more studies are needed to examine which environmental-level determinants may be most important [29-31]. Environmental-level factors that were found to be related to dietary behaviors are physical environmental-level factors, such as the availability at home [32-35] and perceived availability in the neighborhood [36,37], and economic environmental-level factors, such as the perception of price [7,34,36]. Environmental-level factors have shown to be more important (eg, price [8,38]) and more unfavorable [7,34,35] among lower-educated people. Because computer-tailored interventions are traditionally targeted at individual-level factors, environmental-level factors are only addressed to a limited extent and mostly in the form of perceived barriers to be overcome. Modern technology makes it possible to provide more sophisticated feedback on environmental-level factors, such as objectively assessed availability of healthy products in the local food environment. Adding this type of feedback can potentially increase the efficacy of computer-tailored nutrition education interventions because an important category of determinants is addressed. The efficacy of adding environmental-level feedback on the availability and prices of healthy food products and the arrangement of the home food environment in computer-tailored interventions has yet to be established because there is no existing evidence so far. There is, however, some evidence from the physical activity domain, but the evidence for the additional value of environmental-level feedback is still inconclusive [39-42].

To test the added value of targeting environmental-level factors in a computer-tailored nutrition education intervention, we developed 2 versions of a Web-based computer-tailored nutrition education intervention. One version addresses individual cognitions and self-regulation processes (the basic version) and the other version additionally addresses environmental-level factors (the plus version). The main aim of this study was to establish the efficacy of both intervention versions at 1- and 4-months postintervention on the intake of fruit, vegetables, high-energy snacks, and fat, compared to generic nutrition information. The efficacy is evaluated in both the total study sample and among people who do not comply with the guidelines for fruit, vegetables, high-energy snacks, or fat at baseline because these people should particularly benefit from the intervention. Another aim was to explore potential educational differences in intervention effects.

We hypothesized that both intervention versions would result in a larger increase in fruit and vegetable intake and a larger decrease in high-energy snack and fat intake compared to generic nutrition information and that the effects would be more prominent in the risk groups. In addition, we hypothesized that the intervention version that targets environmental-level factors would be more effective for lower-educated participants than for higher-educated participants because environmental-level factors are suggested to be more strongly related to behavior among lower-educated people.

## Methods

### Overview

A detailed description of the study protocol has been published elsewhere [43]; therefore, a summary of the methodology and protocol is described subsequently. The trial was registered in the Dutch Trial Registry (NTR3396) and was approved by the Medical Ethics Committee of the Erasmus Medical Centre in Rotterdam, the Netherlands (NL35430.078.11 / MEC-2010-408).

### Study Design

A 3-group randomized controlled trial (RCT) was conducted from March 2012 to December 2013 in the Netherlands. Participants were randomly assigned to the basic intervention group (n=456), the plus intervention group (n=459), or the control group (n=434). Fruit, vegetable, high-energy snack, and fat intake were assessed at baseline (T0), 1-month postintervention (T1), and 4-months postintervention (T2). The whole study was conducted online.

### Study Procedure

#### Participants

**Figure 1:**
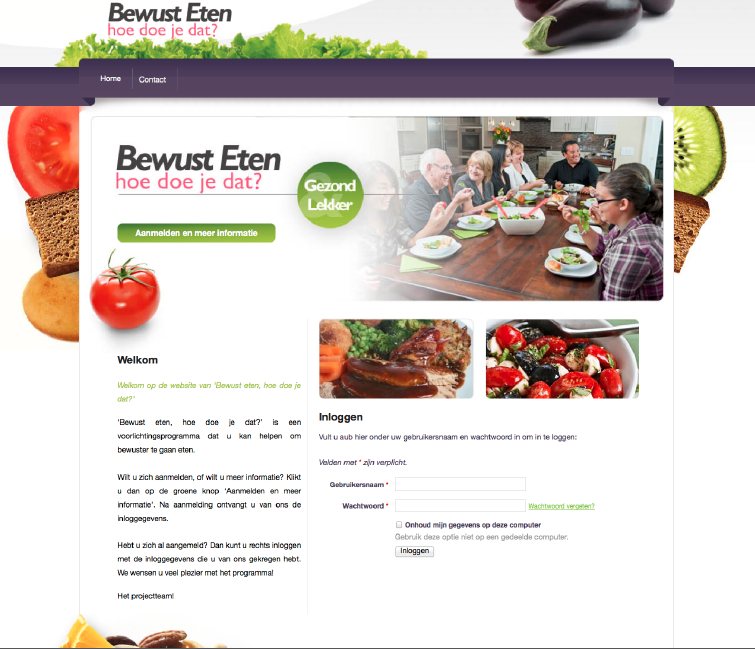
Screenshot of the study website.

The target group for this trial were adults aged 20 to 65 years. A power calculation [43] (power=.80; significance level α=.05) showed that 1400 participants would be sufficient to detect a small intervention effect (ES<0.30). To account for dropout between each measurement and a potential higher dropout among the lower educational group, 2000 people needed to be recruited. Participants were recruited between March and October 2012 from the general population in 5 cities in the South of the Netherlands, primarily via personal mailings sent to 26,402 random home addresses. These addresses were obtained via municipalities. Additionally, Facebook advertisements, advertisements in local newspapers, local television, and promotion activities in shopping malls (ie, distribution of flyers and talking to people) were used for recruitment. People received a flyer with information about the goal, procedure, and incentives for the study. Participants could sign up for the study by phone, email, or via the study website (Figure 1). Participants were included in the study if they were aged between 20 and 65 years, had a sufficient understanding of the Dutch language (in reading and writing), and had access to a computer that was connected to the Internet. Exclusion criteria were being on a diet prescribed by a physician or dietician, having a medical condition that implied restrictions in eating behavior (eg, CVD or bowel disease), and not willing to sign an informed consent.

#### Procedure

**Figure 2:**
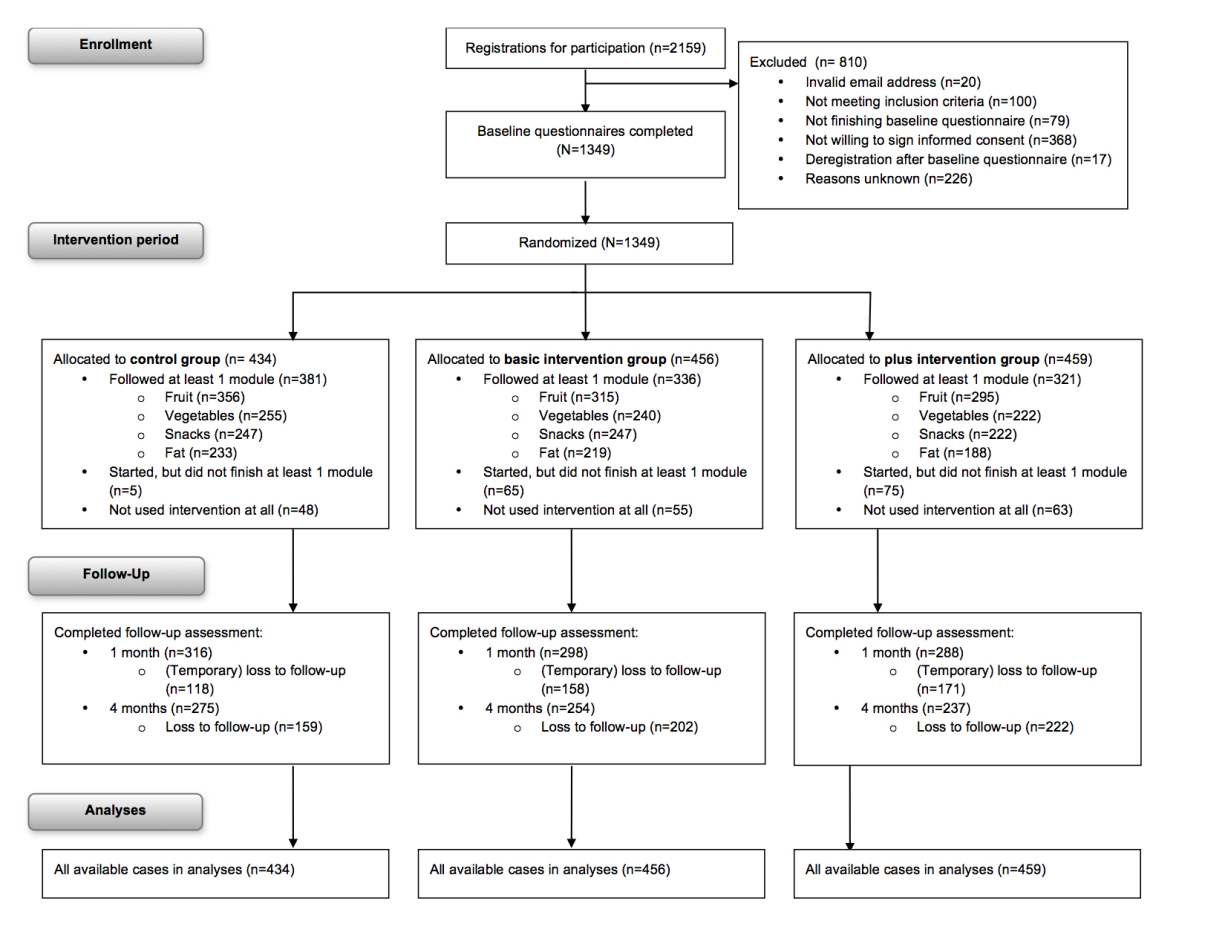
Overview of the study procedure and measurements.

After signing up for the study, a link to the online baseline questionnaire was sent via email. One email reminder to fill out the baseline questionnaire was sent 2 weeks after the initial invitation. The baseline questionnaire started with assessing the inclusion and exclusion criteria. People who met the inclusion criteria were asked to give online informed consent before they could continue with the baseline questionnaire. Additionally, a written informed consent form was sent via postal mail or email and people were asked to sign and return the form. Only people who signed and returned the written form were included in the study. One month after completing the baseline questionnaire, participants could start with the intervention program. Randomization took place just before participants received the invitation to access the website. Individual participants were randomly assigned to 1 of the study conditions in a computer-determined sequence. Participants received a log-in code and password through email, which gave them access to the allocated intervention program on the study website (Figure 1). Participants were asked to visit the website at least 3 times during a 2-month period. Email reminders to visit or revisit the intervention were sent every 2 weeks. At 1 and 4 months after the 2-month intervention period, participants were asked by email to fill out online questionnaires again. Email reminders were sent 2 and 4 weeks after the initial invitation. Twenty iPads and 500 gift vouchers of €20 were allotted among participants who completed all questionnaires. To improve the response, 1 extra iPad and 25 extra gift vouchers were allotted for filling out the first and second follow-up questionnaires, respectively. The study procedure, including the enrollment of participants and the distribution of the questionnaires and interventions, is shown in Figure 2.

### Intervention

#### Overview

The objective of the Web-based computer-tailored nutrition education intervention [43] was to increase fruit and vegetable intake and decrease high-energy snack and saturated fat intake. The 2 intervention versions were developed in a systematic way following the steps of the Intervention Mapping protocol [44] and were partly based on existing interventions [45,46]. Both versions consisted of 4 modules (ie, fruit, vegetables, high-energy snacks, and fat), each containing 3 sessions that could be worked through during 6 consecutive weeks. Two weeks after each intervention visit, email reminders were sent to prompt returning to the intervention to evaluate progress toward achieving the behavioral goal or to receive feedback on another target behavior. Completion of the entire intervention took approximately 160 minutes. The first session took approximately 20 to 30 minutes to complete per module, and the second and third sessions approximately 10 to 20 minutes per module. The information was written at grade level 4-6 (ie, years of education) to make the information comprehensible for lower-educated people as well. The intervention was delivered via a website that participants could log into. A pretest among both higher- (n=45) and lower-educated (n=20) people showed that both intervention versions were appreciated by the target group and that the information was usable and comprehensible, but there was also some room for improvement. Based on this pretest, some small adaptations were made (eg, decreasing the length of the text). Both intervention versions are described briefly subsequently, but a detailed description is published elsewhere [43].

#### Basic and Plus Versions of the Intervention

Both intervention versions were based on self-regulation theory [47], the Theory of Planned Behavior [48], and the Precaution Adoption Process Model [49], and targeted knowledge, awareness, intention, attitude, self-efficacy, goal setting, and action and coping planning. All 4 modules had a similar structure, except for the fat module that did not contain methods to target attitude and self-efficacy in the first session to limit participant burden because the assessment of fat intake was quite long. The 3 sessions were arranged according to the self-regulation phases preaction, action, and evaluation of the behavior change [50,51].

Participants could choose which behavior(s) they wanted to receive feedback and guidance on. After choosing a target behavior, the first session started with providing information to increase knowledge about the chosen behavior [52]. Subsequently, participants could assess their behavior. Based on this assessment, tailored personal, normative, and comparative feedback was provided to increase awareness [44]. Attitude was targeted by providing feedback on self-selected advantages and disadvantages [44,53]. Feedback on self-selected potential barriers and difficult situations was provided to increase self-efficacy [44,52,53]. At the end of the first session, participants could set a goal and formulate an implementation intention for when, where, and how to make the behavior change [44,52,54-56]. After the first session, participants could start enacting their plans and initiate performing their new behavior for 2 weeks.

The second and third sessions provided the opportunity to evaluate the progress of the behavior change. Participants first monitored their goal achievement in the past week and were provided with feedback on their progress [52,57,58]. When the goal was not achieved, attitude and self-efficacy were targeted to stimulate participants to take a second attempt. All participants were stimulated to formulate coping plans for expected difficult situations [56]. If necessary, goals could be adapted to make them more achievable or more challenging. The third session additionally provided information on how to maintain the behavior change over time. This information was based on the 3 self-regulation phases [50,51] and described the different self-regulation steps participants could follow (eg, “what to do when your plan isn’t successful?” or “what can you do when you relapse to your old habit?”).

#### Plus Version of the Intervention

The content of the plus version was identical to the basic version, but the first session additionally included environmental-level feedback on the availability and prices of healthy food products in the supermarket where the participant usually does his or her shopping and on the availability and location of food products in the home food environment. The second and third sessions were identical to the basic version.

Before receiving feedback on dietary intake levels, participants could state for each target behavior at which supermarket they buy their food products (eg, fruit). The tailored feedback that was provided contained the availability and price of products in this specific supermarket. The feedback on the availability and price of food products in the specific supermarket was incorporated into the feedback on attitude and self-efficacy. After selecting relevant disadvantages or barriers (eg, “fruit is expensive”), participants received objective environmental-level information presented as a list of selected food products available in the supermarket, with the price of the products if relevant for the disadvantage or barrier. This environmental-level feedback was also provided in a separate section. Before stating a goal and action plan, participants could review the list with the availability and prices of selected food products in their supermarket, relevant for the target behavior (eg, in the module on fruit, only information about fruit was provided). Subsequently, participants could use this information to set goals and make action plans. The availability and prices of selected food products were collected by observing participating supermarkets (n=31) in the 5 cities where the study was conducted. For supermarkets that did not provide permission for these observations (n=27), more general information on availability and prices for this supermarket was provided. The information on availability was based on information that was available via websites or flyers of the supermarket, if possible (n=13). When no information was available on websites or in flyers (n=14), general information on availability of the selected food products (ie, which products are available in most supermarkets) was provided to the participants. For price, only general information (ie, which products are usually least expensive in supermarkets) was provided for supermarkets that did not provide permission for observations.

In addition, the arrangement of the home food environment was targeted. Participants could fill out whether they always have fruit, vegetables, or high-energy snacks available at home and where they store fruit, vegetables, or high-energy snacks. Subsequently, participants received feedback on possible improvements in availability and storage of products (eg, “make sure you always have fruit available and store the fruit in a visible place, like in a fruit bowl”). Participants could use this information to create a more supportive home environment. This feedback consisted of approximately 8 to 10 lines of text. This section about the home food environment was incorporated in the intervention before the objective information on availability and prices in supermarkets.

Because of the extra information that was provided in the first session of the plus version, the extra time to work through the first session of this version took approximately 5 to 10 minutes per module (ie, 20 to 40 minutes extra when the whole intervention was used).

#### Control Condition

The generic information for the control group also consisted of 4 modules, each consisting of 3 sessions that could be worked through in 6 consecutive weeks. Participants could choose which behavior(s) they wanted to get information about and received nontailored information about fruit, vegetables, high-energy snacks, and/or saturated fat, which was derived from general information that is available from the Netherlands Nutrition Centre [59] and the Dutch Vegetable and Fruit Centre [60]. For example, information was provided about the importance of complying with guidelines, how people can eat more fruit, and how people can maintain eating less fat. The control program had the same name and was provided via the same website and in the same layout as the intervention. Similar reminders for visiting or revisiting the program were sent to the participants.

### Measures

#### Overview

Online questionnaires were used to collect self-reported data on the intake of fruit, vegetables, high-energy snacks, and saturated fat.

#### Vegetable and Fruit Intake

Vegetable and fruit intake were measured with a validated food frequency questionnaire [61,62]. Four items were used to measure vegetable intake in average grams per day. Participants were asked on how many days per week they usually consume cooked and raw vegetables or salads (ranging from 0-7 days per week) and how many tablespoons of cooked and raw vegetables or salads they usually eat on these days (ranging from 1 to ≥6). One tablespoon of cooked vegetables represented 50 grams of vegetables and 1 tablespoon of raw vegetables or salads represented 25 grams of vegetables. Grams of vegetables per day were calculated by multiplying the frequency by the amount of tablespoons multiplied by grams and dividing by 7 (days a week).

Six items were used to assess fruit intake in average number of pieces of fruit per day. Participants were asked on how many days per week they usually consume citrus fruit, other fruit, or (unsweetened) fruit juices (ranging from 0-7 days per week), and how many pieces of citrus fruit and other fruit or glasses of fruit juices they usually consume on these days (1 to ≥7). Daily amount of fruit was calculated by multiplying the frequency by the amount of pieces or glasses of juice and dividing by 7 (days a week).

#### Fat Intake

Saturated fat intake was measured with a validated food frequency questionnaire (the “fat list”) aimed to assess the frequency and quantity of a variety of food items eaten in the past week [63]. Participants were asked to report on how many days per week they usually consume a selection of food items during or between meals. If applicable, the quantity and kind of products (eg, low-fat or full-fat milk) were also assessed. Based on this questionnaire, fat points were calculated, which represent grams of saturated fat. The total “fat score” was based on 35 questions, assessing food products in the following categories: dairy products (n=11), butter (n=1), gravy (n=3), sandwich fillings (n=6), meat and cheese eaten at dinner (n=4), and snacks (n=10). Based on the frequency and amount of intake and the kind of product, fat points were assigned for each product group, ranging from zero (lowest fat intake) to a maximum of 2-5 (highest fat intake, depending on how much fat a product group contains). The fat points for each product group were summed up to create a total fat points measure. In total, a maximum of 80 fat points could be obtained.

#### Intake of High-Energy Snacks

To measure snack intake, the questions on frequency of high-energy snack intake from the fat list questionnaire [63] were used, in combination with extra items to measure the number of snacks eaten per occasion. A total of 21 items measured high-energy snack intake, such as fried products, candy bars, cookies, and chocolate. High-energy snack intake was calculated as the mean number of high-energy snacks eaten per day by multiplying the frequency per week by the quantity and dividing by 7 (days a week).

#### Demographic Factors

Sex (male vs female), age (in years), place of residence (What is your place of residence?: Heerlen, Roermond, Weert, Venlo, Venray), ethnicity, and educational level were assessed in the baseline questionnaire. To assess educational level, participants had to indicate their highest attained educational level [64]. Educational level was first divided into 3 groups: high educated (higher vocational education and university), moderate educated (intermediate vocational education and higher secondary or preuniversity education), and low educated (no education to lower general secondary education). Because differences in intake levels between low- and moderate-educated people are reported to be small [4], educational level was dichotomized into 2 groups: (0) high educated and (1) lower educated (low and moderate educated). Ethnicity (non-Western and Western) was defined according to the procedures of Statistics Netherlands [65]; a participant was considered to be of Western ethnicity if both parents were born in Europe (except for Turkey), North America, Oceania, Indonesia, or Japan. If at least 1 parent was born elsewhere, the participant was considered to be of non-Western ethnicity.

### Statistical Analyses

Descriptive statistics were used to describe the study groups at baseline. Multiple logistic regression analyses were conducted to test for selective dropout from the study. Demographics (ie, gender, age, ethnicity, educational level, place of residence), study group, and intake of fruit, vegetables, high-energy snacks, and fat at baseline were regressed on dropout (yes=1; no=0) at first and second follow-up measurements. To study equality of the study groups at baseline, 3 multiple logistic regression analyses were conducted with study group as dependent variable and age, gender, ethnicity, educational level, place of residence, and intake of fruit, vegetables, high-energy snacks, and fat at baseline as independent variables.

Repeated measures analyses were conducted to study the intervention effects on the intake of fruit, vegetables, high-energy snacks, and saturated fat. General linear mixed models with time as a repeated statement and an unstructured covariance structure were used to study differences in changes over time between the 3 study groups (group×time interaction). Using a linear mixed model allowed for inclusion of cases with missing data; therefore, all randomized participants are included [66]. No clustering of observations of participants within cities was found so including place of residence as an extra level was not indicated. The linear mixed model analyses were only adjusted for place of residence.

Separate analyses were conducted for the 4 outcome measures. In each analysis time, group, and a group×time interaction were entered as independent variables and the group×time interactions were interpreted. Place of residence and variables that differed between 2 or more groups at baseline or that were predictors for dropout were entered as covariates. The type III Wald test was used to test overall statistical significance of the group×time effects. When this overall test statistic for the group×time interaction (ie, *F* test) was significant (*P*≤.05), in-depth results for group differences were examined and reported (ie, unstandardized regression coefficients that represent the difference in change over time between 2 groups). Both intervention groups were compared to the control group and to each other. To make comparisons with previous studies and between different intervention modules, ES were calculated by dividing the unstandardized regression coefficient representing the difference in change over time between 2 groups by the square root of the variance at the corresponding time point (eg, unstandardized regression coefficient/√variance_T1_ for T1). An ES <0.30 was considered small, an ES between 0.30 and 0.80 was considered moderate, and an ES >0.80 was considered large [67].

Before running the main analyses, we explored for each outcome measure whether educational level moderated the intervention effects, by adding a group×time×education interaction term to the repeated measures analyses. If these interaction terms were statistically significant (*P*≤.05), stratified analyses were conducted.

The repeated measures analyses were conducted in both the total sample and the risk groups for each dietary outcome (ie, participants who, at baseline, consumed <200 grams of vegetables, <2 pieces of fruit, >2 pieces of high-energy snacks, or did not comply with gender- and age-specific guidelines for fat intake).

Depending on the distribution of the outcome variable, the original or the log-transformed value was used in the repeated measures analyses. All tests were 2-sided and alpha levels were set at .05. All analyses were performed with SPSS version 22.0 (IBM Corp, Armonk, NY, USA).

## Results

### Participant Characteristics at Baseline

A total of 1349 participants were included in the analyses. The mean age of the participants was 49.05 years (SD 10.62), 35.43% (478/1349) were male, 1.34% (18/1348) had a non-Western ethnic background, and 45.66% (616/1349) were in the high-educated group (Table 1). The mean daily vegetable intake was 159.12 grams (SD 69.24), daily fruit intake was 1.85 pieces (SD 1.29), daily high-energy snack intake was 3.34 pieces (SD 2.98), and mean saturated fat intake was 17.91 fat points (SD 6.07). The sizes of the risk groups were as follows: 1014 participants (75.17%) did not comply with the recommendation of 200 grams of vegetables a day, 803 participants (59.53%) did not comply with the guideline of 2 pieces of fruit per day, 808 participants (59.90%) consumed more than 2 high-energy snacks per day, and 627 participants (46.48%) did not comply with the age- and gender-specific guidelines for maximum fat intake. The plus group was significantly younger than the control group (OR 0.99, 95% CI 0.97-0.99, *P*=.04) and the plus group consisted of more lower-educated people than the basic group (OR 1.33, 95% CI 1.01-1.74, *P*=.04). Hence, age and education were included in the repeated measures analyses as covariates in addition to place of residence.

**Table 1:** Participant characteristics at baseline.

Characteristic			Total n=1349	Control n=434	Basic n=459	Plus n=456	Group comparison, OR (95% CI)^a^		
							Basic vs control	Plus vs control	Plus vs basic
**Background characteristics**									
	Age (years), mean (SD)		49.05 (10.62)	50.01 (10.40)	48.63 (11.10)	48.54 (10.30)	0.99 (0.98, 1.00)	0.99 (0.97, 0.99)	0.99 (0.99, 1.01)
	**Gender, n (%)**								
		Male	478 (35.43)	145 (33.4)	165 (36.2)	168 (36.6)	1.00	1.00	1.00
		Female	871 (64.57)	289 (66.6)	291 (63.8)	291 (63.4)	0.80 (0.60, 1.08)	0.80 (0.59, 1.07)	1.00 (0.75, 1.34)
	**Ethnicity (n=1348), n (%)**								
		Western	1330 (98.66)	425 (98.2)	451 (98.9)	454 (98.9)	1.00	1.00	1.00
		Non-Western	18 (1.34)	8 (1.8)	5 (1.1)	5 (1.1)	0.50 (0.16, 1.60)	0.49 (0.16, 1.55)	0.98 (0.28, 3.44)
	**Educational level, n (%)**								
		High	616 (45.66)	184 (42.4)	232 (50.9)	200 (43.6)	1.00	1.00	1.00
		Lower	733 (54.34)	250 (57.6)	224 (49.1)	259 (56.4)	0.77 (0.58, 1.01)	1.02 (0.77, 1.34)	1.33 (1.01, 1.74)
	**Place of residence (ie, cities in the Netherlands), n (%)**								
		Heerlen	323 (23.94)	103 (23.7)	113 (24.8)	107 (23.3)	1.00	1.00	1.00
		Roermond	217 (16.09)	69 (15.9)	78 (17.1)	70 (15.3)	1.04 (0.68, 1.59)	1.00 (0.65, 1.54)	0.95 (0.62, 1.44)
		Weert	251 (18.60)	77 (17.7)	82 (18.0)	92 (20.0)	1.01 (0.67, 1.54)	1.19 (0.79, 1.79)	1.17 (0.79, 1.75)
		Venlo	304 (22.54)	104 (24.0)	93 (20.4)	107 (23.3)	0.82 (0.56, 1.22)	1.01 (0.69, 1.49)	1.23 (0.83, 1.81)
		Venray	254 (18.83)	81 (18.7)	90 (19.7)	83 (18.1)	1.05 (0.70, 1.59)	1.04 (0.68, 1.57)	0.98 (0.65, 1.46)
**Dietary intake**									
	**Vegetable intake (grams)**								
		Mean (SD)	159.12 (69.24)	157.73 (64.54)	162.68 (72.76)	156.91 (69.94)	1.00 (0.99, 1.00)	1.00 (0.99, 1.00)	1.00 (0.99, 1.00)
		Not complying, n (%)	1014 (75.17)	330 (76.0)	338 (74.1)	346 (75.4)			
	**Fruit intake (pieces)**								
		Mean (SD)	1.85 (1.29)	1.80 (1.23)	1.92 (1.36)	1.81 (1.27)	1.07 (0.97, 1.20)	1.03 (0.92, 1.15)	0.95 (0.85, 1.05)
		Not complying, n (%)	803 (59.53)	261 (60.1)	263 (57.7)	279 (60.8)			
	**High-energy snack intake (pieces)**								
		Mean (SD)	3.34 (2.98)	3.19 (2.74)	3.30 (2.94)	3.51 (3.24)	1.04 (0.98, 1.10)	1.05 (0.99, 1.11)	1.02 (0.96, 1.07)
		Not complying, n (%)	808 (59.90)	251 (57.8)	275 (60.3)	282 (61.4)			
	**Saturated fat intake (fat points) (n=1348)**								
		Mean (SD)	17.91 (6.07)	17.99 (6.07)	17.60 (6.09)	18.13 (6.05)	0.98 (0.96, 1.01)	0.99 (0.96, 1.02)	1.01 (0.98, 1.04)
		Not complying, n (%)	627 (46.47)	197 (45.4)	203 (44.5)	227 (49.5)			

^a^ Logistic regression model with age, gender, ethnicity, educational level, place of residence, fruit intake, vegetable intake, high-energy snack intake, and fat intake as independent variables.

### Loss to Follow-Up

A total of 1349 participants filled out the baseline questionnaire, 902 participants filled out the complete first follow-up questionnaire (33.14% dropout), and 766 participants completely filled out the second follow-up questionnaire (43.22% dropout) (Figure 2). Younger participants were more likely to drop out between baseline and T1 (OR 1.02, 95% CI 1.01-1.03, *P*=.002) and T2 (OR 1.03, 95% CI 1.02-1.04, *P*<.001) compared to older participants. Dropout between baseline and T1 was higher in the basic (34.6%, 158/456; OR 1.42, 95% CI 1.07-1.90, *P*=.02) and the plus group (37.3%, 171/459; OR 1.55, 95% CI 1.17-2.07, *P*=.003) than in the control group (27.2%, 118/434). Dropout between baseline and T2 was also higher in the basic (44.3%, 202/456; OR 1.36, 95% CI 1.04-1.80, *P*=.03) and plus group (48.4%, 222/459; OR 1.58, 95% CI 1.20-2.07, *P=*.001) than in the control group (36.6%, 159/434).

### Assessment of Moderation by Educational Level

No significant interaction effects with educational level were found in the total sample and risk groups for fruit, vegetable, and saturated fat intake. Therefore, stratified analyses by educational level were not indicated for these behaviors. For high-energy snack intake, the interaction with education was borderline significant in both the total sample (*F*_4,932.32_=2.34, *P*=.053) and risk group (*F*_4,533.59_=2.37, *P*=.051). Therefore, stratified analyses by educational level were performed as additional analyses for high-energy snack intake.

### Intervention Effects

#### Fruit Intake

Even though fruit intake increased over time (*F*_2,953.39_=38.44, *P*<.001), there was no difference in change over time between the 3 groups (*F*_4,1151.24_=1.09, *P*=.36) in the total sample (Tables 2 and 3).

Among participants who consumed less than 2 pieces of fruit at baseline (n=803), a significant difference in change over time was found between the groups (*F*_4.523.71_=3.61, *P*=.007). The plus group had a significantly larger increase in fruit intake than the control group, between baseline and both T1 (plus vs control: ES =0.25, *P*=.01) and T2 (plus vs control: ES =0.37, *P*=.001). At medium term, the plus version also resulted in a larger increase in fruit intake than the basic version (plus vs basic: ES =0.22, *P*=.04) (Tables 4 and 5).

**Table 2:** Estimated marginal means at baseline and 1- and 4-month follow-ups and changes over time for the total sample (N=1349).^a^

Time point		Fruit (pieces)		Vegetables (grams)		High-energy snacks (pieces)		Saturated fat (fat points)	
		Mean (SE)	Mean change (95% CI)^b^	Mean (SE)	Mean change (95% CI)^b^	Mean (SE)	Mean change (95% CI)^b,c^	Mean (SE)	Mean change (95% CI)^b^
**Baseline**									
	Control	1.81 (0.06)	NA	158.93 (3.31)	NA	3.21 (0.14)	NA	17.96 0.29)	NA
	Basic	1.94 (0.06)	NA	163.24 (3.22)	NA	3.29 (0.14)	NA	17.60 (0.29)	NA
	Plus	1.83 (0.06)	NA	158.96 (3.23)	NA	3.50 (0.14)	NA	18.09 (0.29)	NA
**1-month follow-up**									
	Control	2.05 (0.07)	0.24 (0.12, 0.36)	164.30 (3.71)	5.37 (–1.12, 11.86)	3.06 (0.13)	–0.15 (–0.39, 0.09)	17.62 (0.31)	–0.33 (–0.80, 0.14)
	Basic	2.25 (0.07)	0.32 (0.19, 0.44)	180.34 (3.76)	17.10 (10.44, 23.67)	2.42 (0.13)	–0.86 (–1.11, –0.62)	16.17 (0.31)	–1.43 (–1.91, –0.95)
	Plus	2.22 (0.07)	0.39 (0.27, 0.52)	170.63 (3.81)	11.66 (4.92, 18.43)	2.66 (0.13)	–0.84 (–1.08, –0.59)	17.36 (0.31)	–0.73 (–1.21, –0.25)
**4-month follow-up**									
	Control	2.00 (0.08)	0.19 (0.04, 0.33)	158.67 (3.76)	–0.27 (–6.94, 6.41)	2.84 (0.13)	–0.37 (–0.62, –0.12)	17.13 (0.30)	–0.83 (–1.33, –0.33)
	Basic	2.14 (0.08)	0.20 (0.05, 0.34)	173.28 (3.81)	10.03 (3.18, 16.89)	2.50 (0.13)	–0.79 (–1.04, –0.53)	15.84 (0.31)	–1.77 (–2.82, –1.25)
	Plus	2.17 (0.08)	0.35 (0.20, 0.50)	167.61 (3.92)	8.66 (1.60, 15.72)	2.50 (0.13)	–1.00 (–1.26, –0.74)	16.67 (0.31)	–1.42 (–1.95, –0.89)

^a^ Based on linear mixed model including place of residence, age, education, study group, time, and group×time.

^b^ As compared to T0.

^c^ Significance tests based on natural logarithm of high-energy snacks.

**Table 3:** Results of linear mixed model analyses for the total sample (N=1349).

Outcome measure			*F* (*df*)	B^a^ (95% CI)	*P*	ES
**Fruit intake**						
	Group×time×education^b^		0.49 (4, 1015.26)	—	.74	—
	Group×time^c^		1.09 (4, 1151.24)	—	.36	—
	Time^d^		38.44 (2, 953.39)	—	<.001	—
	**Differences in change over time after 1 month (T1)**					
		Basic vs control^c^	—	NA	NA	NA
		Plus vs control^c^	—	NA	NA	NA
		Plus vs basic^c^	—	NA	NA	NA
	**Differences in change over time after 4 months (T2)**					
		Basic vs control^c^	—	NA	NA	NA
		Plus vs control^c^	—	NA	NA	NA
		Plus vs basic^c^	—	NA	NA	NA
**Vegetable intake**						
	Group×time×education^b^		1.40 (4, 904.08)	—	.23	—
	Group×time^c^		1.97 (4, 905.49)	—	.10	—
	Time^d^		16.69 (2, 906.08)	—	<.001	—
	**Differences in change over time after 1 month (T1)**					
		Basic vs control^c^	—	NA	NA	NA
		Plus vs control^c^	—	NA	NA	NA
		Plus vs basic^c^	—	NA	NA	NA
	**Differences in change over time after 4 months (T2)**					
		Basic vs control^c^	—	NA	NA	NA
		Plus vs control^c^	—	NA	NA	NA
		Plus vs basic^c^	—	NA	NA	NA
**High-energy snack intake** ^e^						
	Group×time×education^b^		2.34 (4, 932.32)	—	.053	—
	Group×time^c^		5.77 (4, 933.35)	—	<.001	—
	Time^d^		54.81 (2, 1310.46)	—	<.001	—
	**Differences in change over time after 1 month (T1)**					
		Basic vs control^c^	—	–0.71 (–1.06, –0.37)	<.001	–0.30
		Plus vs control^c^	—	–0.69 (–1.04, –0.34)	.001	–0.29
		Plus vs basic^c^	—	0.03 (–0.32, 0.37)	.36	0.01
	**Differences in change over time after 4 months (T2)**					
		Basic vs control^c^	—	–0.42 (–0.77, –0.06)	.006	–0.18
		Plus vs control^c^	—	–0.63 (–0.99, –0.27)	.002	–0.27
		Plus vs basic^c^	—	–0.21 (–0.58, 0.15)	.75	–0.09
**Saturated fat intake**						
	Group×time×education^b^		1.64 (4, 919.05)	—	.16	—
	Group×time^c^		3.02 (4, 919.65)	—	.02	—
	Time^d^		39.29 (2, 919.57)	—	<.001	—
	**Differences in change over time after 1 month (T1)**					
		Basic vs control^c^	—	–1.10 (–1.77, –0.42)	.001	–0.19
		Plus vs control^c^	—	–0.40 (–1.07, 0.28)	.25	0.07
		Plus vs basic^c^	—	0.70 (0.02, 1.38)	.045	0.12
	**Differences in change over time after 4 months (T2)**					
		Basic vs control^c^	—	–0.94 (–1.66, –0.22)	.01	–0.17
		Plus vs control^c^	—	–0.59 (–1.32, 0.14)	.11	–0.11
		Plus vs basic^c^	—	0.35 (–0.39, 1.09)	.36	0.06

^a^ B=unstandardized regression coefficient for difference in change over time between groups.

^b^ Based on linear mixed model including place of residence, age, education, study group, time, group×time, time×education, group×education, and group×time×education.

^c^ Based on linear mixed model including place of residence, age, education, study group, time, and group×time.

^d^ Based on linear mixed model including place of residence, age, education, study group, and time.

^e^ Repeated measures analyses on natural logarithm of high-energy snacks; estimates based on original variable.

**Table 4:** Estimated marginal means at baseline and at 1- and 4-month follow-ups and changes over time for the risk groups.^a^

Time point		Fruit (pieces) (n=803)		Vegetables (grams) (n=1014)		High-energy snacks (pieces) (n=808)		Saturated fat (fat points) (n=627)	
		Mean (SE)	Mean change (95% CI)^b^	Mean (SE)	Mean change (95% CI)^b^	Mean (SE)	Mean change (95% CI)^b,c^	Mean (SE)	Mean change (95% CI)^b^
**Baseline**									
	Control	1.04 (0.03)	NA	130.53 (2.21)	NA	4.75 (0.19)	NA	22.98 (0.30)	NA
	Basic	1.04 (0.03)	NA	128.99 (2.18)	NA	4.77 (0.18)	NA	22.65 (0.30)	NA
	Plus	1.03 (0.03)	NA	127.27 (2.16)	NA	5.03 (0.18)	NA	22.82 (0.28)	NA
**1-month follow-up**									
	Control	1.56 (0.07)	0.52 (0.38, 0.66)	146.11 (3.66)	16.08 (9.34, 22.82)	4.10 (0.18)	–0.65 (–1.01, –0.28)	21.13 (0.42)	–1.85 (–2.57, –1.13)
	Basic	1.67 (0.08)	0.64 (0.49, 0.78)	157.78 (3.84)	28.79 (21.65, 35.94)	3.26 (0.18)	–1.51 (–1.87, –1.15)	19.35 (0.42)	–3.30 (–4.03, –2.56)
	Plus	1.80 (0.08)	0.78 (0.63, 0.92)	152.72 (3.82)	25.45 (18.35, 32.56)	3.41 (0.18)	–1.62 (–1.98, –1.25)	20.78 (0.41)	–2.04 (–2.75, –1.33)
**4-month follow-up**									
	Control	1.41 (0.08)	0.37 (0.22, 0.52)	143.25 (3.61)	12.72 (6.01, 19.43)	3.80 (0.18)	–0.94 (–1.32, –0.57)	20.55 (0.41)	–2.43 (–3.17, –1.69)
	Basic	1.56 (0.09)	0.53 (0.36, 0.69)	153.20 (3.79)	24.21 (17.11, 31.32)	3.32 (0.18)	–1.46 (–1.83, –1.08)	18.83 (0.42)	–3.82 (–4.57, –3.60)
	Plus	1.80 (0.09)	0.77 (0.60, 0.94)	148.03 (3.86)	20.76 (13.53, 28.00)	3.28 (0.19)	–1.75 (–2.14, –1.37)	19.58 (0.43)	–3.24 (–4.03, –2.46)

^a^ Based on linear mixed model including place of residence, age, education, study group, time, and group×time.

^b^ As compared to T0.

^c^ Significance tests based on natural logarithm of high-energy snacks.

**Table 5:** Results of linear mixed model analyses for the risk groups.

Outcome measure			*F* (*df*)	B^a^ (95% CI)	*P*	ES
**Fruit intake (n=803)**						
	Group×time×education^b^		0.64 (4, 522.34)	—	.64	—
	Group×time^c^		3.61 (4, 523.71)	—	.007	—
	Time^d^		136.31 (2, 523.62)	—	<.001	—
	**Differences in change over time after 1 month (T1)**					
		Basic vs control^c^	—	0.12 (–0.08, 0.32)	.25	0.11
		Plus vs control^c^	—	0.26 (0.06, 0.46)	.01	0.25
		Plus vs basic^c^	—	0.14 (–0.06, 0.35)	.18	0.12
	**Differences in change over time after 4 months (T2)**					
		Basic vs control^c^	—	0.16 (–0.07, 0.38)	.16	0.14
		Plus vs control^c^	—	0.40 (0.18, 0.63)	.001	0.37
		Plus vs basic^c^	—	0.25 (0.01, 0.48)	.04	0.22
**Vegetable intake (n=1014)**						
	Group×time×education^b^		1.21 (4, 679.32)	—	.31	—
	Group×time^c^		2.15 (4, 671.86)	—	.07	—
	Time^d^		72.11 (2, 654.00)	—	<.001	—
	**Differences in change over time after 1 month (T1)**					
		Basic vs control^c^	—	NA	NA	NA
		Plus vs control^c^	—	NA	NA	NA
		Plus vs basic^c^	—	NA	NA	NA
	**Differences in change over time after 4 months (T2)**					
		Basic vs control^c^	—	NA	NA	NA
		Plus vs control^c^	—	NA	NA	NA
		Plus vs basic^c^	—	NA	NA	NA
**High-energy snack intake** ^e^ **(n=808)**						
	Group x time x education^b^		2.37 (4, 533.59)	—	.051	—
	Group x time^c^		4.30 (4, 534.96)	—	.002	—
	Time^d^		144.93 (2, 535.40)	—	<.001	—
	**Differences in change over time after 1 month (T1)**					
		Basic vs control^c^	—	–0.86 (–1.38, –0.35)	<.001	-0.34
		Plus vs control^c^	—	–0.97 (–1.49, –0.45)	.001	-0.38
		Plus vs basic^c^	—	–0.11 (–0.62, 0.41)	.90	-0.04
	**Differences in change over time after 4 months (T2)**					
		Basic vs control^c^	—	–0.51 (–1.04, 0.02)	.02	–0.20
		Plus vs control^c^	—	–0.81 (–1.34, –0.28)	.008	–0.32
		Plus vs basic^c^	—	–0.30 (–0.83, 0.24)	.74	–0.12
**Saturated fat intake (n=627)**						
	Group×time×education^b^		0.90 (4, 425.13)	—	.46	—
	Group×time^c^		2.99 (4, 441.26)	—	.02	—
	Time^d^		112.02 (2, 419.08)	—	<.001	—
	**Differences in change over time after 1 month (T1)**					
		Basic vs control^c^	–	–1.45 (–2.48, –0.42)	.006	–0.28
		Plus vs control^c^	–	–0.19 (–1.20, 0.83)	.72	–0.04
		Plus vs basic^c^	–	1.26 (0.24, 2.28)	.02	0.24
	**Differences in change over time after 4 months (T2)**					
		Basic vs control^c^	—	–1.38 (–2.44, –0.33)	.01	–0.28
		Plus vs control^c^	—	–0.81 (–1.89, 0.27)	.14	–0.16
		Plus vs basic^c^	—	0.58 (–0.51, 1.66)	.30	0.12

^a^ B=unstandardized regression coefficient for difference in change over time between groups.

^b^ Based on linear mixed model including place of residence, age, education, study group, time, group×time, time×education, group×education, and group×time×education.

^c^ Based on linear mixed model including place of residence, age, education, study group, time, and group×time.

^d^ Based on linear mixed model including place of residence, age, education, study group, and time.

^e^ Repeated measures analyses on natural logarithm of high-energy snacks; estimates based on original variable.

#### Vegetable Intake

Vegetable intake increased over time in the total sample (*F*_2,906.08_=16.69, *P*<.001), but there was no difference in change over time between the 3 groups (*F*_4,905.49_=1.97, *P*=.10) (Tables 2 and 3). The same results were found in the risk group: vegetable intake increased over time (*F*_2,654.00_=72.11, *P*<.001), but no difference between the 3 groups was found (*F*_4,671.86_=2.15, *P*=.07)(Tables 4 and 5).

#### High-Energy Snack Intake

High-energy snack intake had a very skewed distribution and, therefore, was log-transformed. The significance tests were based on the log-transformed variable, but the unstandardized regression coefficients and ES were based on the original variable.

In the total sample, there was a significant difference in decrease of high-energy snacks between the groups (*F*_4,933.35_=5.77, *P*<.001). Both intervention groups had a significantly larger decrease in high-energy snack intake than the control group between baseline and both T1 (basic vs control: ES =–0.30, *P*<.001; plus vs control: ES =–0.29, *P*=.001) and T2 (basic vs control: ES =–0.18, *P*=.006; plus vs control: ES =–0.27, *P*=.002) (Tables 2 and 3).

Stratified analyses showed that for lower-educated participants both the basic and plus version resulted in a larger decrease than the control intervention between baseline and T1 (basic vs control: ES =–0.33, *P*<.001; plus vs control: ES =–0.23, *P*=.02). Between baseline and T2, only the basic version resulted in a larger decrease than the control intervention (basic vs control: ES =–0.23, *P*=.009). For high-educated participants, both intervention groups had a larger decrease than the control group between baseline and T1 (basic vs control: ES =–0.31, *P*=.006; plus vs control: ES =–0.38, *P*=.01*).* Between baseline and T2, the plus version resulted in a larger decrease than the control intervention (plus vs control: ES =–0.53, *P*<.001) and basic version (plus vs basic: ES =–0.32, *P*=.04).

Also in the risk group (n=808), significant differences in change over time were found (*F*_4,534.96_=4.30, *P*=.002). Both intervention groups had a significant larger decrease than the control group between baseline and T1 (basic vs control: ES =–0.34, *P*<.001; plus vs control: ES =–0.38, *P*=.001) and T2 (basic vs control: ES =–0.20, *P*=.02; plus vs control: ES =–0.32, *P*=.008) (Tables 4 and 5).

Stratified analyses showed that among lower-educated participants only the basic version resulted in a larger decrease in high-energy snack intake than the control intervention between baseline and both T1 (basic vs control: ES =–0.40, *P*=.002) and T2 (basic vs control: ES =–0.26, *P*=.03). For higher-educated participants, both intervention groups had a larger decrease than the control group between baseline and T1 (basic vs control: ES =–0.34, *P*=.02; plus vs control: ES=–0.50, *P*=.001). Between baseline and T2, the plus version resulted in a larger decrease than the control intervention (plus vs control: ES =–0.64, *P*<.001) and basic version (plus vs basic: ES =–0.38, *P*=.03).

#### Fat Intake

Differences in change over time between the groups were found (*F*_4,919.65_=3.02, *P*=.02) in the total sample. The basic group had a larger decrease in saturated fat intake than the control group between baseline and both T1 (basic vs control: ES =–0.19, *P*=.001) and T2 (basic vs control: ES =–0.17, *P*=.01). In addition, the decrease between baseline and T1 was significantly smaller for the plus group compared to the basic group (plus vs basic: ES =0.12, *P*=.045) (Tables 2 and 3).

Also in the risk group (n=627), differences in change over time between the groups were found (*F*_4,441.26_=2.99, *P*=.02). The basic group had a significantly larger decrease in saturated fat intake than the control group between baseline and both T1 (basic vs control: ES=–0.28, *P*=.006) and T2 (basic vs control: ES=–0.28, *P*=.01). Between baseline and T1, the decrease was lower for the plus group than for the basic group (plus vs basic: ES =0.24, *P*=.02) (Tables 4 and 5).

## Discussion

### Principal Findings

An RCT was conducted to evaluate the short- (1 month) and medium-term (4 months) efficacy and educational differences in efficacy of a cognitive (basic) and an environmental feedback (plus) version of a Web-based computer-tailored nutrition education intervention [43] on self-reported intake of fruit, vegetables, high-energy snacks, and saturated fat compared to a generic nutrition information control group, in both the total sample and risk groups that did not comply with guidelines for fruit, vegetables, high-energy snacks, or fat at baseline.

In both the total sample and risk group, the basic version was more effective than generic nutrition information in changing saturated fat intake; in the short term, this version was also more effective than the plus version. The plus version was more effective than generic nutrition information in changing fruit intake among participants who did not comply with guidelines for fruit intake. At the medium term, this version was also more effective in improving fruit intake than the basic version. Both intervention versions were not more effective than generic nutrition information in increasing vegetable intake. Both intervention versions were effective in decreasing high-energy snack intake in both the total sample and risk group, although educational differences were found. In the short term, the basic version was effective among both high- and lower-educated participants and the plus version was effective among lower-educated participants in the total sample and high-educated participants in both the total sample and risk group. In the medium term, however, only the basic version was effective for lower-educated participants, whereas for high-educated participants the plus version was more effective than both the control intervention and basic version. No educational differences were found for the other behaviors indicating that the intervention can be equally effective among high- and lower-educated people. As expected, the effects were more prominent and ES were slightly larger in risk groups, which is an important finding because people who do not comply with dietary guidelines are most in need of improving their diets. These results show that, except for fruit intake in the risk group and high-energy snack intake among high-educated participants, the plus intervention version did not clearly outperform the basic version.

The results of this study add favorably to the evidence base of positive effects of Web-based computer-tailored nutrition education interventions on fruit and fat intake [10,13-15] and more prominent effects in risk groups [17,45,68]. The results on vegetable intake, however, compare unfavorably to those of multiple previous studies [10,13-15]. Finding effects among lower-educated participants is also in-line with previous studies on computer-tailored interventions [16,17]. This was the first computer-tailored intervention with a module on reducing high-energy snack intake. It is promising that both versions showed effects in reducing high-energy snack intake. This can be an important addition to interventions that aim to prevent overweight or obesity.

The plus version was most effective for fruit intake among participants who did not comply with guidelines for fruit intake and, among high-educated participants, for high-energy snack intake. For vegetable and saturated fat intake, however, no effects of the plus version were found. Compared to vegetable and fat intake, fruit and high-energy snack intake can be more easily changed without adapting complete dietary patterns. Therefore, it may be clearer which products can be purchased, making it easier to integrate and apply the information about availability and prices in supermarkets. The home food environment may also be easier to rearrange for high-energy snacks and fruit (eg, by having these products available at home less or more often or storing the products in invisible or visible places, respectively).

The environmental feedback component included feedback on availability of healthy food products and feedback and suggestions to rearrange the home food environment. As a result, it is not clear whether the (additional) effects of the plus version were caused by supermarket information or feedback on the home food environment or by a combination of both. Incorporating environmental-level information about availability and prices in supermarkets was very time-consuming because all supermarkets had to be observed to collect and update the data [43] making large-scale implementation of this version difficult. However, providing feedback on the arrangement of the home food environment is feasible. Future research on the mediating variables of the plus version of the intervention may provide insight into the potential of only targeting the home food environment.

We expected that targeting environmental-level factors would be more effective for lower-educated participants than for high-educated participants since environmental-level factors are suggested to be more important among lower-educated people [8,38]. However, no educational differences were found for fruit, vegetables, and fat. Moreover, for high-energy snacks, more and larger effects of the plus version were found for high-educated participants than for lower-educated participants. One explanation may be that high-educated people are better able to process information [69]; therefore, they may be better able to integrate and apply the environmental-level information, making the plus version more effective for this group. The plus version contained some more information than the basic version. The information on the availability and prices of food products in the supermarkets was provided in clear tables and the feedback on the home food environment contained only 8 to 10 lines of text. However, it may be that the plus version became too long to provide added effects among lower-educated participants.

Because we additionally included self-regulation processes and environmental-level factors, we expected to achieve somewhat larger ES compared to previous studies [10,13]. Although self-regulation processes are suggested to be important to target in interventions [22,23], the results of this study showed that, compared to generic nutrition information, ES of targeting self-regulation processes in addition to individual cognitions were still mostly small. Important components of self-regulation that were included in the intervention are goal setting and action planning. Incorporating goal setting and action planning tools in Web-based computer-tailored interventions is challenging. Goals and plans are most effective when they are challenging and specific (ie, when they are of high quality) [70,71]. This requires guidance, which is difficult to provide in Web-based computer-tailored interventions. In our intervention, some guidance was provided by explicitly asking the components of goals (ie, how many products do you want to consume more or less?) and plans (ie, when and how) and by providing short instructions on how to formulate goals and plans. However, to fit with self-regulation, participants formulated their own goals and plans [72]. This may have resulted in goals and plans of low quality, as is found in another Web-based computer-tailored intervention [73]. Goals and plans of low quality may decrease the effects of self-regulation on behavior. Providing more insight into incorporating goal setting and action planning tools in Web-based computer-tailored interventions may increase efficacy.

Environmental-level factors are suggested to play an important role in dietary behavior [7,27,28,32-37]. The results of this study, however, indicate that additionally targeting these factors as was done in this intervention does not largely increase the efficacy compared to generic nutrition information or only targeting individual cognitions and self-regulation processes, except for fruit intake in the risk group and high-energy snack intake among high-educated participants. Although ES were slightly larger for the plus version than for the basic version for fruit and high-energy snack intake, these differences were not always significant. In addition, for saturated fat intake, the plus version was less effective than the basic version in the short term. One explanation may be that the provided environmental information was not extensive enough to have an additional effect. The home food environment, for example, was only a small part of the intervention, whereas adapting the home food environment is a complex behavior that includes multiple behavioral determinants, such as awareness and self-efficacy. Although we included all supermarkets in the selected cities, food products, especially snacks and high-fat products, may be bought in other stores, such as train or gas stations. In addition, for supermarkets that were not willing to participate, only very general information about the availability and prices in supermarkets was provided in the intervention. If we would have been able to provide more sophisticated environmental-level feedback, the added effects of the plus version may have been larger.

Another reason for finding small ES may be the nonoptimal exposure to the different modules and sessions, which often happens in Internet interventions [73,74]. In addition, participants could choose their own target behavior(s) and were consequently not necessarily exposed to all modules. Exposure was highest for the fruit module (it appeared first in the list of 4 modules) and lowest for the fat module (it appeared last in the list of 4 modules). Exposure also differed between study groups and was higher in the control group than in the intervention groups. One reason for this difference in exposure may be that the participant burden of the control version was lower than for both intervention versions because participants in the control group only had to read information and did not have to fill out assessment questionnaires and formulate goals and plans.

### Limitations

When interpreting the results of this study, some limitations should be taken into account. We may have recruited a selective sample of the population due to selective response. Intake levels were more favorable compared to the general Dutch population [4], which may indicate that our study population was more motivated for or interested in healthy nutrition. However, because no intake differences were found between the study groups at baseline, the results are not expected to be biased by confounding variables. Despite our efforts to overrecruit low-educated participants (eg, by spreading extra flyers in low socioeconomic neighborhoods), response was selective according to educational level and only 19.3% (261/1349) of the study sample were low educated. Because intake differences between low and moderate educational groups are reported to be small [4], low- and moderate-educated participants were combined into 1 group. Consequently, intervention effects among very low-educated people are unknown. In addition to selective response, there was high dropout and dropout was selective for age and study group. A high dropout is not uncommon in Web-based computer-tailored interventions [24,74-76], but it may have influenced the intervention effects. By conducting linear mixed model analyses and by correcting the analyses for predictors for dropout, an attempt was made to minimize bias potentially caused by dropout. The selective sample and high and selective dropout may have decreased the external validity of the results. Therefore, the results are only generalizable to Dutch adults who are interested in healthy eating (but who can still improve dietary intake patterns). According to our power calculation, 1400 participants were needed to detect small intervention effects between the intervention groups and control group. Although fewer participants were included in the study than initially planned, we were still able to include 1349 available cases in the analyses. The effects for the differences between the 2 intervention groups, however, need to be interpreted with caution because the study was not powered to detect these differences. The differences in ES between both intervention versions were, however, quite small. Therefore, probably no other conclusions would be drawn when more people were included in the study and power was larger. Another component that may have influenced the intervention effects is the difference in length between both intervention versions. Although validated questionnaires were used to measure fruit, vegetable, and saturated fat intake, the study relied on self-reported data. This may be less valid than using more objective instruments, such as biomarkers. Effects based on self-reported intake levels may not be seen when using biomarker validation as was demonstrated in a study by Kroeze et al [77]. Using such objective instruments was not feasible in this trial because of the large number of participants, but future studies should verify the effects that were found in the present study using biomarkers of intake. The questionnaires are, however, suitable to rank people according to their intake levels and according to changes and differences in intake levels [61,63]. The items to measure high-energy snack intake were derived from validated questionnaires. These items are also used in previous studies to measure high-energy snack intake (eg, [24,78]). Although the method used to assess high-energy snack intake is also used in validated questionnaires to measure intake levels and products were derived from a validated questionnaire to measure (saturated) fat intake, validity and reliability of these items to measure the amount of snacks eaten per day are not known; therefore, these results should be interpreted with caution. In addition, the questionnaires were validated for hard-copy use only. However, because all 3 study groups filled out the same questionnaires, bias has probably been minimized.

### Conclusions

The Web-based computer-tailored intervention targeting individual cognitions and self-regulation processes was effective in decreasing self-reported high-energy snack and saturated fat intake. Additionally targeting environmental-level factors was effective in increasing self-reported fruit intake in the risk group and high-energy snack intake. The intervention effects were more prominent among people who did not comply with dietary guidelines. Equal intervention effects were found for both higher- and lower-educated participants, except for high-energy snack intake for which additionally targeting environmental-level factors was most effective among high-educated participants.

No additional effects of also targeting environmental-level factors were found for self-reported saturated fat intake and, among lower-educated people, for self-reported high-energy snack intake. In addition, providing environmental-level information is time-consuming. Therefore, the basic intervention version may be more feasible for large-scale implementation for these dietary behaviors. For high-energy snack intake among high-educated people and fruit intake, however, additionally targeting the arrangement of the home food environment and the perception of the availability and prices should be considered.

## Multimedia Appendix 1

CONSORT EHEALTH checklist V1.6.2 [79].

## Abbreviations

CVD: cardiovascular diseases
ES: effect size(s)
RCT: randomized controlled trial
