# Supplementary material for: Short- and Medium-Term Efficacy of a Web-Based Computer-Tailored Nutrition Education Intervention for Adults Including Cognitive and Environmental Feedback: Randomized Controlled Trial
Source: J Med Internet Res. 2015 Jan 19;17(1):e23. doi: 10.2196/jmir.3837 (PMC4319071; doi:10.2196/jmir.3837)
Supplement: Supplementary file 1 [file jmir_v17i1e23_app1.pdf]

CONSORT-EHEALTH Checklist V1.6.2 Report

(based on CONSORT-EHEALTH V1.6), available at [http://tinyurl.com/consort-ehealth-v1-6].

3837

Date completed

9/3/2014 12:40:56

by

Linda Springvloet

Evaluation of the short and medium term efficacy of a web-based computer-tailored nutrition education intervention for adults including cognitive and environmental feedback: Results of a randomized controlled trial

TITLE

1a-i) Identify the mode of delivery in the title

"[..]web-based computer-tailored nutrition education intervention[..]"

1a-ii) Non-web-based components or important co-interventions in title

Not applicable; there are no non-web-based components in this study.

1a-iii) Primary condition or target group in the title

"[..] for adults [..]"

ABSTRACT

1b-i) Key features/functionalities/components of the intervention and comparator in the METHODS section of the ABSTRACT

"[..]a basic (tailored intervention targeting individual cognitions and self-regulation processes; n=456), plus (basic intervention additionally targeting environmental-level factors; n=459) and control (generic nutrition information; n=434) group."

Both intervention versions were based on self-regulation theory, Theory of Planned Behavior and Precaution Adoption Process Model.

1b-ii) Level of human involvement in the METHODS section of the ABSTRACT

"[..] using online questionnaires."
"[..] computer-tailored [..]."

The whole study was conducted online (intervention and questionnaires).

1b-iii) Open vs. closed, web-based (self-assessment) vs. face-to-face assessments in the METHODS section of the ABSTRACT

"Participants were recruited from the general population [..]."
Methods that were used for recruitment are: personal mailing to random home-addresses, Facebook advertisements, advertisements in (local) newspapers, local television and promotion activities in shopping malls (i.e. distribution of flyers and talking to people).

"[..] using online questionnaires."
The whole study was conducted online.

1b-iv) RESULTS section in abstract must contain use data

"[..] (tailored intervention targeting individual cognitions and self-regulation processes; n=456), plus (basic intervention additionally targeting environmental-level factors; n=459) and control (generic nutrition information; n=434) group."

1b-v) CONCLUSIONS/DISCUSSION in abstract for negative trials

"Both intervention versions were more effective in improving some of the dietary behaviors than generic nutrition information, especially in the risk groups, among both high and lower educated participants. For fruit intake, only the plus version was more effective than providing generic nutrition information. Although feasible, incorporating environmental-level information is time-consuming. Therefore the basic version may be more feasible for further implementation, even though inclusion of feedback on the arrangement of the home food-environment and on availability and prices may be considered for fruit and, for high-educated people, for high-energy snack intake."

INTRODUCTION

2a-i) Problem and the type of system/solution

"To modify dietary intake patterns in large population groups, intervention techniques that can reach large numbers of people and that can be tailored to individual dietary intake patterns are required. Computer tailoring is a suitable technique that can reach a large number of people at relatively low costs [10]. In computer-tailored (CT) nutrition education, health information is adapted to the specific needs and characteristics of a person [11, 12]. Several reviews have shown that (web-based) CT interventions can be effective in improving the intake of fruit, vegetables and fat compared to generic or no information [10, 13-15], also among lower educated people [16, 17]. The effect sizes (ES) of existing CT nutrition education interventions are, however, often small [10, 13, 15]. It is therefore important to find ways to increase the size of the effects, for example by targeting 'new' determinants or behavior change processes."

The intervention that was developed is intended as a stand-alone intervention, aimed at the general adult population.

2a-ii) Scientific background, rationale: What is known about the (type of) system

"Up to now, nutrition education interventions have mainly targeted motivational determinants, such as attitude and self-efficacy. Although motivation is an important first step in the behavior change process, it is not likely that motivation alone will lead to sustained behavior change [18-20]. This approach neglects, for example, important volitional [18, 20] and self-regulation processes, such as goal setting and action planning, which focus on translating intention into action and facilitate actual changes.
Self-regulation skills, like planning and monitoring, are shown to predict dietary behavior [21] and using intervention techniques that foster self-regulation, such as goal setting and providing feedback on performance, is associated with larger improvements in dietary outcomes [22]. In addition, a meta-regression of Michie and colleagues showed that including the self-regulatory technique self-monitoring of behavior in combination with other self-regulation behavior change techniques (i.e. prompt intention formation, prompt specific goal setting, provide feedback on performance and prompt review of behavioral goals) is likely to increase the efficacy of interventions aimed at promoting healthy nutrition [23]. Self-regulation processes like goal setting and action planning were shown to be feasible to apply in interventions targeting, for example, weight maintenance [24], weight-loss among young adults [25] and Diabetes management [26], but the additional effect of targeting self-regulation processes in CT interventions has to be established yet.
In addition, there is increasing evidence for the role of environmental-level factors as important drivers of (dietary) behaviors [27, 28]. Since CT interventions are traditionally targeted at individual-level factors, environmental-level factors are only addressed to a limited extent and mostly in the form of perceived barriers that have to be overcome. Modern technology makes it possible to provide more sophisticated feedback on environmental-level factors, such as objectively assessed availability of healthy products in the local food-environment. Adding this type of feedback can potentially increase the efficacy of CT nutrition education interventions, since by doing so, an important category of determinants is addressed. Important environmental-level factors that were found to be related to dietary behaviors are physical environmental-level factors, such as the availability at home [29-32] and perceived availability in the neighborhood [33, 34], and economic environmental-level factors, such as the perception of price [7, 31, 33]. Environmental-level factors have shown to be more important (e.g. price [8, 35]) and more unfavorable [7, 31, 32] among lower educated people. The efficacy of adding environmental-level feedback on the availability and prices of healthy food products and the arrangement of the home food-environment in CT interventions has yet to be established, as there is no existing evidence so far. There is, however, some evidence from the physical activity domain, but the evidence for the additional value of environmental-level feedback is still inconclusive [36-39]."

METHODS

3a) CONSORT: Description of trial design (such as parallel, factorial) including allocation ratio

"The main aim of this study was to establish the efficacy of both intervention versions at one and four-months post-intervention on the intake of fruit, vegetables, high-energy snacks and fat, compared to generic nutrition information. The efficacy is evaluated in both the total study sample as well as among people who do not comply with the guidelines for fruit, vegetables, high-energy snacks or fat at baseline, since these people should particularly benefit from the intervention. Another aim was to explore potential educational differences in intervention effects.
We hypothesized that both intervention versions would result in a larger increase in fruit and vegetable intake and a larger decrease in high-energy snack and fat intake compared to generic nutrition information and that the effects would be more prominent in the risk groups. In addition, we hypothesized that the intervention version that targets environmental-level factors would be more effective for lower educated participants than for high-educated participants, since environmental-level factors are suggested to be stronger related to behavior among lower educated people."

3b) CONSORT: Important changes to methods after trial commencement (such as eligibility criteria), with reasons

"One extra iPad and 25 extra gift vouchers were allotted for filling out the first- and second follow-up questionnaire respectively, in order to improve the response."

No changes were made to the intervention, website or study procedure.

3b-i) Bug fixes, Downtimes, Content Changes

Not applicable; no changes to the intervention or website were made after the start of the trial.

4a) CONSORT: Eligibility criteria for participants

"Participants were included in the study if they were between 20 and 65 years, had a sufficient understanding of the Dutch language (in reading and writing) and had access to a computer that is connected to the Internet. Exclusion criteria were: being on a diet prescribed by a physician or dietician, having a medical condition that implies restrictions in eating behavior (e.g. CVD or bowel disease) and not willing to sign an informed consent."

4a-i) Computer / Internet literacy

"[..] had access to a computer that is connected to the Internet."

4a-ii) Open vs. closed, web-based vs. face-to-face assessments:

"Participants could sign up for the study by phone, e-mail or via the study website (Figure 1). [..] Participants received a login code and password through e-mail, which gave them access to the allocated intervention program on the study website (Figure 1)."
"The whole study was conducted online."
"Participants were recruited between March and October 2012 from the general population in five cities in the South of the Netherlands, mainly via personal mailings sent to 26,402 random home-addresses. These addresses were obtained via municipalities. Additionally, Facebook advertisements, advertisements in (local) newspapers, local television and promotion activities in shopping malls (i.e. distribution of flyers and talking to people) were used for recruitment. People received a flyer with information about the goal, procedure and incentives for the study."

Participants were not completely anonymous; participants had to provide their name and e-mailadress during the registration process.

4a-iii) Information giving during recruitment

"People received a flyer with information about the goal, procedure and incentives for the study."

"People who met the inclusion criteria were asked to give online informed consent before they could continue with the baseline questionnaire. Additionally, a written informed consent form was sent via postal- or e-mail and people were asked to sign and return the form. Only people who signed and returned the written form were included in the study."

4b) CONSORT: Settings and locations where the data were collected

"[..] in five cities in the South of the Netherlands."
"The whole study was conducted online."

4b-i) Report if outcomes were (self-)assessed through online questionnaires

"Online questionnaires were used to collect self-reported data on the intake of fruit, vegetables, high-energy snacks and fat."

4b-ii) Report how institutional affiliations are displayed

5) CONSORT: Describe the interventions for each group with sufficient details to allow replication, including how and when they were actually administered

5-i) Mention names, credential, affiliations of the developers, sponsors, and owners

Not applicable.

5-ii) Describe the history/development process

We conducted a pretest / formative evaluation in another city in the south of the Netherlands. This is described in detail in a paper that describes the study protocol (http://www.biomedcentral.com/1471-2458/14/47).

5-iii) Revisions and updating
Not applicable; no major changes were made during the evaluation process.
5-iv) Quality assurance methods
Not applicable.
5-v) Ensure replicability by publishing the source code, and/or providing screenshots/screen-capture video, and/or providing flowcharts of the algorithms used
A screenshot of the intervention website is provided and the intervention is described in the methods section. The intervention is described in more detail in a paper that describes the study protocol (http://www.biomedcentral.com/1471-2458/14/47). More information about the intervention can be obtained from the authors.
5-vi) Digital preservation
Currently, the website (and intervention) cannot be accessed by others. It is, however, archived by the authors.
5-vii) Access
"Participants received a login code and password through e-mail, which gave them access to the allocated intervention program on the study website (Figure 1). Participants were asked to visit the website at least three times during a two-month period. E-mail reminders to (re-)visit the intervention were sent every two weeks. One and four months after the two-month intervention period, participants were asked by e-mail to fill out online questionnaires again. E-mail reminders were sent two and four weeks after the initial invitation. Twenty iPad's and 500 gift vouchers of 20 Euros were allotted among participants who completed all questionnaires. To improve the response, one extra iPad and 25 extra gift vouchers were allotted for filling out the first and second follow-up questionnaire respectively."
5-viii) Mode of delivery, features/functionalities/components of the intervention and comparator, and the theoretical framework
The intervention (e.g. theoretical framework and content) is described in the methods section. A more detailed description can be found in the study protocol (http://www.biomedcentral.com/1471-2458/14/47).
5-ix) Describe use parameters
"Participants were asked to visit the website at least three times during a two-month period. E-mail reminders to (re-)visit the intervention were sent every two weeks. One and four months after the two-month intervention period, participants were asked by e-mail to fill out online questionnaires again."
5-x) Clarify the level of human involvement
"The whole study was conducted online."

Participants received invitations and reminders by e-mail for both using the (control) intervention and filling out the questionnaires:
"After signing up for the study, a link to the online baseline questionnaire was sent via e-mail. One e-mail reminder for filling out the baseline questionnaire was sent two weeks after the initial invitation."
"Participants received a login code and password through e-mail [..]"
"E-mail reminders to (re-)visit the intervention were sent every two weeks.
"One and four months after the two-month intervention period, participants were asked by e-mail to fill out online questionnaires again. E-mail reminders were sent two and four weeks after the initial invitation."
5-xi) Report any prompts/reminders used
"One e-mail reminder for filling out the baseline questionnaire was sent two weeks after the initial invitation. [..] E-mail reminders to (re-)visit the intervention were sent every two weeks. [..] participants were asked by e-mail to fill out online questionnaires again. E-mail reminders were sent two and four weeks after the initial invitation."
5-xii) Describe any co-interventions (incl. training/support)
Not applicable.
6a) CONSORT: Completely defined pre-specified primary and secondary outcome measures, including how and when they were assessed
"Online questionnaires were used to collect self-reported data on the intake of fruit, vegetables, high-energy snacks and fat."
"Fruit, vegetable, high-energy snack and fat intake were assessed at baseline (T0), one-month post-intervention (T1) and four-months post-intervention (T2)."

In the methods section, it is described how these outcome measures (and demographics) were assessed and calculated.
6a-i) Online questionnaires: describe if they were validated for online use and apply CHERRIES items to describe how the questionnaires were designed/deployed
" [..] the questionnaires were validated for hard-copy use only."

The questionnaires are, however, used in multiple studies to examine the efficacy of web-based computer-tailored nutrition education interventions.
6a-ii) Describe whether and how "use" (including intensity of use/dosage) was defined/measured/monitored
Exposure to the intervention (i.e. whether participants started the intervention and which module they followed) was based login rates, derived from server registrations.

6a-iii) Describe whether, how, and when qualitative feedback from participants was obtained
Participants were able to contact us by e-mail.
6b) CONSORT: Any changes to trial outcomes after the trial commenced, with reasons
Not applicable.
7a) CONSORT: How sample size was determined
7a-i) Describe whether and how expected attrition was taken into account when calculating the sample size
"A power calculation [40] (power=0.80; significance level α=.05) showed that 1,400 participants would be sufficient to detect a small intervention effect (ES<0.30). To account for dropout between each measurement and a potential higher dropout among the lower educational group, 2,000 people needed to be recruited."
7b) CONSORT: When applicable, explanation of any interim analyses and stopping guidelines
Not applicable.
8a) CONSORT: Method used to generate the random allocation sequence
"Randomization took place just before participants received the invitation to access the website. Individual participants were randomly assigned to one of the study conditions in a computer-determined sequence."
8b) CONSORT: Type of randomisation; details of any restriction (such as blocking and block size)
No block or cluster randomization was applied.

"Individual participants were randomly assigned to one of the study conditions in a computer-determined sequence."

9) CONSORT: Mechanism used to implement the random allocation sequence (such as sequentially numbered containers), describing any steps taken to conceal the sequence until interventions were assigned
"Participants received a login code and password through e-mail, which gave them access to the allocated intervention program on the study website (Figure 1)." This procedure was the same for the control group. The control group also received a program, in order to conceal allocation to the control group:
"The control program had the same name and was provided via the same website and in the same layout as the intervention. Similar reminders for (re-) visiting the program were sent to the participants.
10) CONSORT: Who generated the random allocation sequence, who enrolled participants, and who assigned participants to interventions
The sequence was computer-determined. The first author and a research associate enrolled participants and mailed login codes and passwords to the participants.
11a) CONSORT: Blinding - If done, who was blinded after assignment to interventions (for example, participants, care providers, those assessing outcomes) and how
11a-i) Specify who was blinded, and who wasn't
Participants were blinded.
11a-ii) Discuss e.g., whether participants knew which intervention was the "intervention of interest" and which one was the "comparator"
Participants did not know to which study group they were allocated. The procedures in all study groups were the same. Participants did not know which interventions were the interventions of interest.
11b) CONSORT: If relevant, description of the similarity of interventions
"The control program had the same name and was provided via the same website and in the same layout as the intervention. Similar reminders for (re-) visiting the program were sent to the participants."
12a) CONSORT: Statistical methods used to compare groups for primary and secondary outcomes
"Repeated measures analyses were conducted to study the intervention effects on the intake of fruit, vegetables, high-energy snacks and fat. General linear mixed models with 'time' as a repeated statement and an unstructured covariance structure were used to study differences in changes over time between the three study groups ('group X time' interaction). Using a linear mixed model allows for inclusion of cases with missing data and therefore includes all randomized participants [63]. Separate analyses were conducted for the four outcome measures. In each analysis 'time', 'group' and a 'group X time' interaction were entered as independent variables and the 'group X time' interactions were interpreted. Place of residence and variables that differed between two or more groups at baseline or that were predictors for dropout were entered as covariates. The type III Wald test was used to test overall statistical significance of the 'group X time' effects. When the 'group X time' interaction was significant ($P \leq .05$), in-depth results for group differences were examined and reported. Both intervention groups were compared to the control group and to each other. In addition, an ES was calculated by dividing the unstandardized regression coefficient representing the difference in change over time between two groups by the square root of the variance at the corresponding time-point (e.g. unstandardized regression coefficient/√(varianceT1) for T1). An ES below .30 was considered small, an ES between .30 and .80 was considered moderate and an ES above .80 was considered large [64].
Before running the main analyses we explored for each outcome measure whether educational level moderated the intervention effects, by adding a 'group X time X education' interaction term to the repeated measures analyses. If these interaction terms were statistically significant ($P \leq .05$), stratified analyses were conducted."
12a-i) Imputation techniques to deal with attrition / missing values
Alle randomized participants were included in the analysis. Participants with missing data on the follow-up measurements were also included in the analyses: "Repeated measures analyses were conducted to study the intervention effects on the intake of fruit, vegetables, high-energy snacks and fat. General linear mixed models with 'time' as a repeated statement and an unstructured covariance structure were used to study differences in changes over time between the three study groups ('group X time' interaction). Using a linear mixed model allows for inclusion of cases with missing data and therefore includes all randomized participants [63]."
12b) CONSORT: Methods for additional analyses, such as subgroup analyses and adjusted analyses
Not applicable.

RESULTS

13a) CONSORT: For each group, the numbers of participants who were randomly assigned, received intended treatment, and were analysed for the primary outcome
This data is provided in Figure 2 (a flow-chart).
13b) CONSORT: For each group, losses and exclusions after randomisation, together with reasons
This data is provided in Figure 2 (a flow-chart).
13b-i) Attrition diagram
This data is included in Figure 2 (a flow-chart).
14a) CONSORT: Dates defining the periods of recruitment and follow-up
"Participants were recruited between March and October 2012 [..]"
"A three-group randomized controlled trial (RCT) was conducted from March 2012 to December 2013 in the Netherlands."
"One month after completing the baseline questionnaire participants could start with the intervention program."
"One and four months after the two-month intervention period, participants were asked by e-mail to fill out online questionnaires again."
14a-i) Indicate if critical "secular events" fell into the study period
Not applicable.
14b) CONSORT: Why the trial ended or was stopped (early)
Not applicable.
15) CONSORT: A table showing baseline demographic and clinical characteristics for each group
This data is provided in Table 1 (Participant characteristics at baseline).
15-i) Report demographics associated with digital divide issues
Demographics such as age, gender, ethnicity and education are provided in Table 1 (Participant characteristics at baseline).
16a) CONSORT: For each group, number of participants (denominator) included in each analysis and whether the analysis was by original assigned groups
16-i) Report multiple "denominators" and provide definitions
This data is provided in Figure 2 (a flow-chart).
16-ii) Primary analysis should be intent-to-treat
The analyses are intent-to-treat (all randomized participants are included in the analyses).

No secondary analyses are conducted.
17a) CONSORT: For each primary and secondary outcome, results for each group, and the estimated effect size and its precision (such as 95% confidence interval)

The results of the primary outcome measures (estimated marginal means, unstandardized regression coefficient, effect size, p-value) are shown in Table 2, Table 3, Table 4 and Table 5.

17a-i) Presentation of process outcomes such as metrics of use and intensity of use

The data on intervention use are presented in Figure 2 (a flow-chart).

17b) CONSORT: For binary outcomes, presentation of both absolute and relative effect sizes is recommended

Not applicable; no binary outcomes are examined.

18) CONSORT: Results of any other analyses performed, including subgroup analyses and adjusted analyses, distinguishing pre-specified from exploratory

Not applicable; no subgroup analyses are performed.

18-i) Subgroup analysis of comparing only users

Not applicable; no subgroup analyses are conducted.

19) CONSORT: All important harms or unintended effects in each group

Not applicable.

19-i) Include privacy breaches, technical problems

Not applicable. No incidents were reported.

19-ii) Include qualitative feedback from participants or observations from staff/researchers

Not applicable.

DISCUSSION

20) CONSORT: Trial limitations, addressing sources of potential bias, imprecision, multiplicity of analyses

20-i) Typical limitations in ehealth trials

Multiple potential limitations are acknowledged:

"When interpreting the results of this study some limitations should be taken into account. We may have recruited a selective sample of the population due to selective response. Intake levels were more favorable compared to the general Dutch population [4], which may indicate that our study population was more motivated for or interested in healthy nutrition. However, since no intake differences were found between the study groups at baseline, the results are not expected to be biased by confounding variables. Despite our efforts to over-recruit low-educated participants, for example by spreading extra flyers in low socio-economic neighborhoods, response was selective according to educational level and only 19.3% (261/1,349) of the study sample were low-educated. Because intake differences between low and moderate educational groups are reported to be small [4], low- and moderate-educated participants were combined into one group. Consequently, intervention effects among (very) low-educated people are unknown. In addition to selective response, the (high) dropout was selective for age and intervention group. A high dropout [24, 71-73] is often reported in other web-based CT interventions. By including all randomized participants in the analyses and correcting the analyses for predictors for dropout, an attempt to minimize bias potentially caused by dropout was made. According to our power calculation, 1,400 participants were needed to detect small intervention effects between the intervention groups and control group. Although fewer participants were included in the study than initially planned, we were still able to include 1,349 available cases in the analyses. The effects for the differences between the two intervention groups, however, need to be interpreted with caution, since the study was not powered to detect these differences. The differences in ES between both intervention versions were, however, quite small. Therefore, probably no other conclusions would be drawn when more people were included in the study and power was larger. Although validated questionnaires were used, the study relied on self-reported data. This may be less accurate than using more objective instruments, such as biomarkers, and may have resulted in, for example, response bias or over-reporting. Using objective instruments was, however, not feasible in this trial, because of the large number of participants. The questionnaires are, however, suitable to rank people according to their intake levels and according to changes and differences in intake levels [58, 60]. In addition, the questionnaires were validated for hard-copy use only. However, since all three study groups filled out the same questionnaires, bias has probably been minimized."

21) CONSORT: Generalisability (external validity, applicability) of the trial findings

21-i) Generalizability to other populations

"We may have recruited a selective sample of the population due to selective response. Intake levels were more favorable compared to the general Dutch population [4], which may indicate that our study population was more motivated for or interested in healthy nutrition. However, since no intake differences were found between the study groups at baseline, the results are not expected to be biased by confounding variables. Despite our efforts to over-recruit low-educated participants, for example by spreading extra flyers in low socio-economic neighborhoods, response was selective according to educational level and only 19.3% (261/1,349) of the study sample were low-educated. Because intake differences between low and moderate educational groups are reported to be small [4], low- and moderate-educated participants were combined into one group. Consequently, intervention effects among (very) low-educated people are unknown. In addition to selective response, the (high) dropout was selective for age and intervention group. A high dropout [24, 71-73] is often reported in other web-based CT interventions. By including all randomized participants in the analyses and correcting the analyses for predictors for dropout, an attempt to minimize bias potentially caused by dropout was made."

21-ii) Discuss if there were elements in the RCT that would be different in a routine application setting

Not applicable.

22) CONSORT: Interpretation consistent with results, balancing benefits and harms, and considering other relevant evidence

22-i) Restate study questions and summarize the answers suggested by the data, starting with primary outcomes and process outcomes (use)

The discussion starts with the study questions and a summary of the results.

"A RCT was conducted to evaluate the short (1 month) and medium (4 months) term efficacy and educational differences in efficacy of a cognitive (basic) and an environmental-feedback (plus) version of a web-based CT nutrition education intervention [40] on the intake of fruit, vegetables, high energy snacks and fat compared to a generic nutrition information control group, in both the total sample and risk groups that did not comply with guidelines for fruit, vegetables, high-energy snacks or fat at baseline.

In both the total sample and risk group, the basic version was more effective than generic nutrition information in changing fat intake and at the short term this version was also more effective than the plus version. The plus version was more effective than generic nutrition information in changing fruit intake among participants who did not comply with guidelines for fruit intake. At the medium term this version was also more effective in improving fruit intake than the basic version. Both intervention versions were not more effective than generic nutrition information in increasing vegetable intake. Both intervention versions were effective in decreasing high-energy snack intake in both the total sample and risk group, although educational differences were found. On the short term, the basic version was effective among both high- and low/moderate-educated participants and the plus version was effective among lower educated participants in the total sample and high-educated participants in both the total sample and risk group. On the medium term, however, only the basic version was effective for lower educated participants, while for high-educated participants the plus version was more effective than both the control intervention and basic version. No educational differences were found for the other behaviors, indicating that the intervention can be equally effective among high and low/moderate-educated people. As expected, the effects were more prominent and ES were slightly larger in risk groups, which is an important finding since people who do not comply with dietary guidelines are most in need of improving their diets. These results show that, except for fruit intake in the risk group and high-energy snack intake among high-educated participants, the plus intervention version did not clearly outperform the basic version."

22-ii) Highlight unanswered new questions, suggest future research

"Future research on the mediating variables of the plus version of the intervention may provide insight into the potential of only targeting the home food-environment."

"Providing more insight into incorporating goal setting and action planning tools in web-based CT interventions may increase efficacy."

Other information

23) CONSORT: Registration number and name of trial registry

"Netherlands Trial Registry NTR3396."

24) CONSORT: Where the full trial protocol can be accessed, if available

Dutch Trial registry: http://www.trialregister.nl/trialreg/admin/rctview.asp?TC=3396

Study protocol: http://www.biomedcentral.com/1471-2458/14/47

25) CONSORT: Sources of funding and other support (such as supply of drugs), role of funders

"Acknowledgements: We would like to thank Karin Hummel for her important role in the evaluation study. The study was funded by ZonMw, the Netherlands Organisation for Health Research and Development (grant number: 200110021)."

X26-i) Comment on ethics committee approval

"The trial [..] is approved by the Medical Ethics Committee of the Erasmus Medical Centre in Rotterdam, the Netherlands (NL35430.078.11 / MEC-2010-408)."

x26-ii) Outline informed consent procedures

"People who met the inclusion criteria were asked to give online informed consent before they could continue with the baseline questionnaire. Additionally, a written informed consent form was sent via postal- or e-mail and people were asked to sign and return the form. Only people who signed and returned the written form were included in the study."

X26-iii) Safety and security procedures

Not applicable. No adverse effects were expected.

X27-i) State the relation of the study team towards the system being evaluated

"Hein de Vries is the scientific director of Vision2Health, a company that licenses evidence-based, innovative, computer-tailored health communication tools. The other authors declare that they have no competing interests."
